# Lecture on Blennorrhagia

**Published:** 1848-01

**Authors:** 


*Lecture on Blennorrhagia.* By M. Ricord. Delivered in the Summer of 1847, at the Hospital du Midi, Paris.

*Continuation of the Causes of Blennorrhagia; Symptoms; Progress; Duration; Complications; Diagnosis; Prognosis; Prophylaxis.*—Sexual intercourse is no doubt the most ordinary mode of transmission in blennorrhagia. In the coïtus we find all the conditions necessary to its developement. It is, however, not indispensable that the tissues should be in a state of sexual excitement for contagion to take place, for it is sufficient that the secretion of one inflamed mucous membrane be deposited on another, to allow this secretion to produce in it an inflammation entirely similar. It is well known that the muco-purulent matter secreted by the urethra, placed on the mucous membrane of the eye, will occasion a blennorrhagic ophthalmia. Another mode of transmission has also been mentioned, but the possibility of it is at present no longer credited. I allude to the story of a jealous husband, who, in order to punish his wife, had contrived to contract blennorrhagia, and attempted to communicate it to his faithless partner, by making her swallow milk in which muco-purulent matter obtained from his own person, had been mixed. We can hardly believe that this muco-purulent matter could, after being absorbed, have been carried, by the circulation, to the genital organs of the woman, there to develope blennorrhagia; no one ever thought of finding out whether the lover who had excited the husband's jealousy might not have been, long before the administration of the treacherous milk, in a state favourable to the transmission of blennorrhagia.

Be this as it may, there are cases mentioned of individuals who accidentally swallowed liquids containing blennorrhagic muco-purulent matter, and who felt no inconvenience therefrom. Hunter, on the contrary, gives one of a child, who, under these circumstances, experienced no ill effects. Indeed, it is extremely difficult to understand how blennorrhagic matter, supposing it even to be admitted, unmixed, into the stomach, could be conveyed to the genital organs by the torrent of the circulation.

Those who have attempted to prove that blennorrhagia is a virulent disease, have brought forward incubation as an argument. Examples of it have been quoted, ranging from twenty-four hours to fifty-two days. But, I may ask, have the patients been closely watched from the first to the fifty second day? Did any one take the trouble of ascertaining in what state those patients were when the disease broke out? No answers are given to these queries, and I conclude, therefore, that such cases are of no value. If I may trespass for a little upon general pathology, I would ask, whether we do not almost constantly notice a certain lapse of time between the application of a cause and its effects, during which time, the symptoms are in some degree concealed? Is there no bronchitis, except when the mucous expectoration is completely established? And yet most medical men will acknowledge the existence of blennorrhagia only when they see a purulent discharge; but what they overlook is, that the disease, before this discharge took place, had passed through a peculiar stage, which I shall describe hereafter.

I admit incubation neither for blennorrhagia nor chancre. With some, the accidents gradually and insensibly arise from the first day, and reach the inflammatory stage after the expiration of five or six. Then the discharge is abundant enough to excite the attention of the patient, or of his medical attendant, and both date the onset of the disease from that moment. With others, the discharge appears the very next day after improper intercourse; but the inflammation mostly takes from one to five days to reach its complete development.—The discharge generally appears on the sixth day.

*Symptoms of Urethral Blennorrhagia considered generally.*—The beginning of this disease is often marked by an exaltation in the functions of the organ, by an exaggeration of its normal sensibility, vitality, and secretion; in a more advanced stage, pain comes on, the secretion disappears, and the surface of the mucous membrane becomes dry; but this state of dryness is soon replaced by a morbid secretion. The affected parts are first moistened by a transparent mucus; a little later, a certain proportion of pus appears, when muco-purulent matter is formed; lastly, pus alone is secreted. The colour is at first a whitish pale; it becomes afterwards straw-colored, or of a darker yellow. When the secretion is very abundant, and contains a few blood-globules, it becomes greenish; and the more the globules increase, the more rusty the green colour will turn. When the pus increases greatly, it assumes a sanious appearance; it may even become sero-sanguineous; but, as a general rule, it may be said that the colour of the secretion varies in proportion to the quantity and quality of the pus. It is important to be acquainted with these different shades, for the knowledge of them may be very useful in diagnosis. I do not mean to say, however, that the green colour is characteristic of virulent blennorrhagia; and I may here mention a case of red blennorrhagia, quoted in an article of the "Dictionnaire de Médecine:" the author of which thinks the red colour was owing to the fact of the disease having been caught from a woman just menstruating. Richerand also thought that virulent blennorrhagia secreted a copper-coloured pus. Some surgeons lay a great stress on the smell of the secretion, and they fancy they can, by means of this character, distinguish a virulent from a simple blennorrhagia. It is true that the genital organs often exhale a shocking smell, which Morgagni very justly compared to the odour emanating from decayed codfish; but this smell has nothing to do with the morbid secretion; it arises solely from uncleanliness in the part, particularly in very fat people.

*Progress of blennorrhagia considered in general.*—We very seldom notice any premonitory symptoms of the disease, such as febrile excitement, or functional disturbance; the inflammation afterwards runs through all its stages to reach a complete cure, or rather, to pass into a chronic state, as we notice in most cases. So, then, we have in the acute form very little tendency to a satisfactory termination, and a very marked one to the chronic state; such are the characters of the usual progress of blennorrhagia. The cure is the more difficult to accomplish, the more concealed the affected parts happen to be;—urethritis, for instance, is more difficult of cure than balano-posthitis. The nature of the functions of the diseased parts contributes very much likewise to retard the cure. Thus we know that the passage of the urine retards the cure of urethritis. Blennorrhagia may begin by the chronic state, and so continue; on the other hand, it sometimes passes from a chronic to an acute state. It may, in general, be said that the acute form is in an inverse ratio to the number of times one individual has been affected with blennorrhagia; the oftener he has had it, the less marked is the acute form. When the disease begins to decline, the secretion alters in character; from being purulent it becomes muco-purulent; then mucous, with a few globules of pus;—but this pus is no longer intimately mixed up with the mucus, as it generally is when the disease is on the increase, but is merely suspended in it, and isolated in a few dots, as it were. The stains on the linen are greyish, with a black dot in the centre. With some people the secretion is very like the leucorrhœal secretion.

*Duration.*—The usual duration of blennorrhagia is from six to eight weeks ; but this law is not an absolute one. There are, to my knowledge, blennorrhagias which, having been caught at the peace of Amiens in 1800, were still going on in 1840.

*Structural alterations.*—As soon as blennorrhagia affects a mucous membrane, it produces alterations in the textures which may be more or less obvious. At first it is a simple effusion followed by alterations in the form, calibre, and capacity of the canal lined by this mucous membrane; plastic changes then take place, and these produce inflammatory congestion, thickening, and hypertrophy of the tissues.

The inflamed parts may become eroded, and these erosions are often followed by real ulcerations. The cicatrix which forms on the ulcers will, of course, produce alterations of form, capacity, consistence, &c., in the parietes of the canal. The granulations may, when they assume a vegetative form, give rise to obstructions. If the congestion persists, it soon degenerates into a hard hypertrophy ; or else, if the plastic lymph which forms it is re-absorbed, the parts become contracted, the cells which contained the plastic matter disappear, the mucous membrane becomes atrophied, thinned, and the result is at last a stricture.

The laws I have just enumerated are those which prevail in general pathology, and we, in fact, see that the inflammations which attack the genito-urinary mucous membrane, have the same consequences as those which affect other mucous membranes.

Some of these consequences have been looked upon as impossible by very good authorities. Hunter, for instance, denies the existence of ulcerations, but he gives only the post-mortem examination of two executed culprits to support his opinion ; it will be quite sufficient for me to state that I have often met with these ulcerations, both in living and in dead subjects. The alterations of tissue which take place in consequence of blennorrhagia, may, in their turn, be the cause of the continuance of certain symptoms, and be the origin of more or less grave accidents. The ulcerations, for example, keep up a purulent discharge. The urine pent up behind strictures assumes irritant properties, and finally produces great alterations in the mucous membrane on which it lies, so as in turn to excite in it an inflammation, followed by a mucous and muco-purulent secretion.

*Accidents which may complicate Blennorrhagia.*—Some of these accidents are inherent to each species of blennorrhagia; I shall mention them in the description of these individual species; others are common to all varieties. Let us now review them :—

1. There is the inflammation of the neighbouring lymphatic vessels and glands. Whatever the seat of blennorrhagia may be, the lymphatics, and after them the glands, may become affected. But these accidents are as frequent in chancre, or in the blennorrhagia symptomatic of chancre, as they are rare in simple blennorrhagia. When they *do* occur in the latter affection, they are simply of an inflammatory character, and easily controlled; the pus which they yield, in the rare cases where suppuration takes place, is not inoculable.

2. Blennorrhagia is very often complicated with abscesses more or less circumscribed, and these are generally situated externally to the mucous membrane, and are never inoculable.

3. Sanguineous exhalation, or hæmorrhage consequent upon a rupture of the urethra.

4. Blennorrhagic ophthalmia: this complication has wrongly been imputed to metastasis, or repercussion.

5. Blennorrhagic arthritis, which has just as erroneously been attributed to these causes.

*Differential Diagnosis considered in general.*—Blennorrhagia does not acknowledge any especial cause, or one which might beforehand be determined; and it is impossible to trace its source by the assistance of symptomatology alone. You see, then, that there is no *virulent* blennorrhagia, save that which is produced by an urethral chancre. And this is the place for unfolding before you the elements of the differential diagnosis, and giving you the means of distinguishing the urethral chancre, first from simple blennorrhagia, and secondly from the discharge symptomatic of secondary accidents.

1. In the diagnosis of chancre we have to consider two orders of symptoms—the one leading to a rational diagnosis of chancre, the other to an absolute one.

*Elements of the Rational Diagnosis.*—The pus is rather of a serous than of a mucous character; it is thin, loaded with organic detritus, rusty, and sanguineous; there is a fixed point where pain, thickening, and a circumscribed induration, exist; the glands of the groin are often enlarged. If the source of the disease be attempted to be traced, these signs will lead to the discovery of a chancre; there is, however, still but a probability of it.

*Absolute Diagnosis.*—The absolute diagnosis is derived from the inspection of the textures, and from inoculation; this latter is an incontestible and uniform proof. The results of inoculation are always positive in the first seven days of the disease, provided it be carefully practised; if the result be then positive, you may be sure that there you have concealed chancre to deal with; if the result be negative, you have to contend merely against a simple, non-virulent discharge. *After* the first seven days, reliance must be placed in inoculation only when the results are positive; but if they are negative, you must not at once conclude that there is no chancre, for in this case the ulcer may have passed the specific stage, and be no longer inoculable.

2. If the blennorrhagia is symptomatic of secondary symptoms, then inoculation gives no results; the only thing to be done is to distinguish it from simple blennorrhagia: herein the diagnosis is very much assisted by the precedents, the coëxistence of other accidents of a secondary nature, and the treatment.

3. In simple blennorrhagia, the only diagnosis to be made is to distinguish between the greater or lesser degree of inflammation, to ascertain the accidents which may complicate it, and the different parts which may be affected.

*Prognosis considered in general.*—The prognosis of blennorrhagia depends on its intensity and its duration; it will also depend on the seat of the disease. We know, for instance, that blennorrhagia will last longer in proportion as the mucous membrane affected is more deeply seated, and thereby rendered more difficult of being exposed. The more an individual has had attacks of blennorrhagia, the more easily will the disease be contracted, and the more difficult it will be to cure it; but the symptoms will always diminish in intensity. The prognosis must vary according to the different species of blennorrhagia.

*Treatment considered in general.*—There is a means which morality condemns, which religion forbids, but which philosophy and hygiene may allow,—I might almost say advise;—there are cases, indeed, when sexual intercourse may be imperative, and yet at the same time there may be the danger of giving or contracting blennorrhagia; here prophylactic means must be had recourse to. *Mediate* coïtus, the male organ being artificially protected by a pellicle, may, indeed, have real advantages during the existence of blennorrhagia. I hardly dare to mention the hygienic means proposed by M. Pigeaux—I mean, masturbation. The mediate coïtus, however, is not always effective as regards preservation from disease; and Madame de Staël was right in saying, when speaking of this means, "It is a breastplate against pleasure, and a cobweb against danger."

*Hygiene.*—Mucous membranes should not be left too long in contact; repetitions and prolongation in the coïtus must be avoided, particularly when there are doubts. It is advisable to wash the parts which are suspected to be impregnated, either with astringents, the urine, or other liquids. On the male side the act ought to be complete and rapid. As the ejaculation carries off any virus that might lie in the urethra, it is a very good cleansing means, as far as this canal is concerned.

So, then, to resume—protection of the organ, complete and rapid coïtus, performed egotistically, ejaculation, micturition, repeated lotions, such are the elements of the prophylactic treatment. After the coïtus, the predisposing causes which have been mentioned should be avoided, particularly beer, asparagus, baths, &c.—*London Lancet.*

